# Rhabdomyolysis-induced acute kidney injury in a cancer patient exposed to denosumab and abiraterone: a case report

**DOI:** 10.1186/s12882-015-0113-6

**Published:** 2015-07-30

**Authors:** Javier A. Neyra, Natalia A. Rocha, Rhea Bhargava, Omkar U. Vaidya, Allen R. Hendricks, Aylin R. Rodan

**Affiliations:** Department of Medicine, University of Texas Southwestern Medical Center, 5323 Harry Hines Blvd, Dallas, TX 75390-8516 USA; Division of Nephrology, University of Texas Southwestern Medical Center, 5323 Harry Hines Blvd, Dallas, TX 75390-8516 USA; Department of Pathology, University of Texas Southwestern Medical Center, 5323 Harry Hines Blvd, Dallas, TX 75390-8516 USA; Department of Medicine, University of Missouri-Kansas City, Kansas City, Missouri USA; Division of Nephrology, University of Missouri-Kansas City, Kansas City, Missouri USA

**Keywords:** Denosumab, Abiraterone, Acute kidney injury, Rhabdomyolysis

## Abstract

**Background:**

Denosumab and abiraterone were approved by the United States Food and Drug Administration in 2011 for the treatment of metastatic castration-resistant prostate cancer. Neither denosumab nor abiraterone is known to cause rhabdomyolysis.

**Case presentation:**

A 76-year-old Caucasian man with metastatic prostate cancer presented with non-oliguric severe acute kidney injury (AKI) 3 weeks after receiving simultaneous therapy with denosumab and abiraterone. The patient had been on statin therapy for more than 1 year with no recent dose adjustments. His physical exam was unremarkable. Blood work on admission revealed hyperkalemia, mild metabolic acidosis, hypocalcemia, and elevated creatine kinase (CK) at 44,476 IU/L. Kidney biopsy confirmed the diagnosis of rhabdomyolysis-induced AKI. The patient responded well to intravenous isotonic fluids and discontinuation of denosumab, abiraterone, and rosuvastatin, with normalization of CK and recovery of kidney function.

**Conclusion:**

We report the first case of biopsy-proven rhabdomyolysis-induced AKI in a cancer patient acutely exposed to denosumab and abiraterone. Whether one of these drugs individually, or the combination, was the bona fide culprit of muscle breakdown is unknown. Nonetheless, our report is hypothesis-generating for further investigations on the effect of these drugs on muscle cells.

## Background

Rhabdomyolysis is characterized by muscle breakdown leading to leakage of sarcoplasmic proteins, electrolytes, and myoglobin into the circulation [1]. Several provoking factors (Table 1) can trigger muscle cell death through direct sarcolemmic injury or depletion of adenosine triphosphate within the myocyte [2]. The final common pathway is an increase in intracellular calcium and consequent protease activation, mitochondrial dysfunction, and production of reactive oxygen species that precipitate muscle cell death [3, 4].

**Table 1:** Identifiable risk factors for rhabdomyolysis

Advanced age^a^
Female gender
Chronic kidney disease^a^
Diabetes mellitus^a^
Hypothyroidism^a^
Inflammatory or metabolic myopathies
Trauma or crush injuries
Hyperthermia
Seizures or muscle tremors
Severe exertion
Prolonged surgical interventions or immobilization
Coma
Sickle cell trait
Hypokalemia
Hypophosphatemia
Severe dehydration
Recreational drugs such as alcohol, cocaine, amphetamines, heroin, phencyclidine
Prescribed drugs such as statins^a^, colchicine, antipsychotics, selective serotonin reuptake inhibitors
Acute viral infections such as Influenza, Coxsackie, EBV, HSV, HIV

^a^Applies to the patient’s case

One of the common complications of rhabdomyolysis is acute kidney injury (AKI). In the US, rhabdomyolysis is the cause of up to 10 % of all cases of AKI [5] and the mortality rate associated with rhabdomyolysis-induced AKI can be as high as 30 %, depending on subjects’ comorbidities [6]. The occurrence of AKI in rhabdomyolysis is likely from a combination of risk factors that include volume depletion, intrarenal vasoconstriction, direct and ischemic proximal tubular injury (myoglobin-driven), and tubular obstruction [7]. The latter mainly occurs in the distal tubules where myoglobin interacts with Tamm–Horsfall protein, particularly in acidic urine [1, 7].

Pharmacologic agents constitute important causes of non-exertional and non-traumatic rhabdomyolysis [6, 8]. Several drugs such as antipsychotics, statins, and selective serotonin reuptake inhibitors have been identified as common culprits of rhabdomyolysis [6, 9], particularly in combination with other patient-specific risk factors.

In 2011, denosumab and abiraterone were approved by the United States Food and Drug Administration for the treatment of metastatic castration-resistant prostate cancer. We present the case of a 76-year-old Caucasian man with a history of metastatic prostate cancer who developed rhabdomyolysis-induced AKI after acute exposure to denosumab and abiraterone.

## Case presentation

**Fig. 1:**
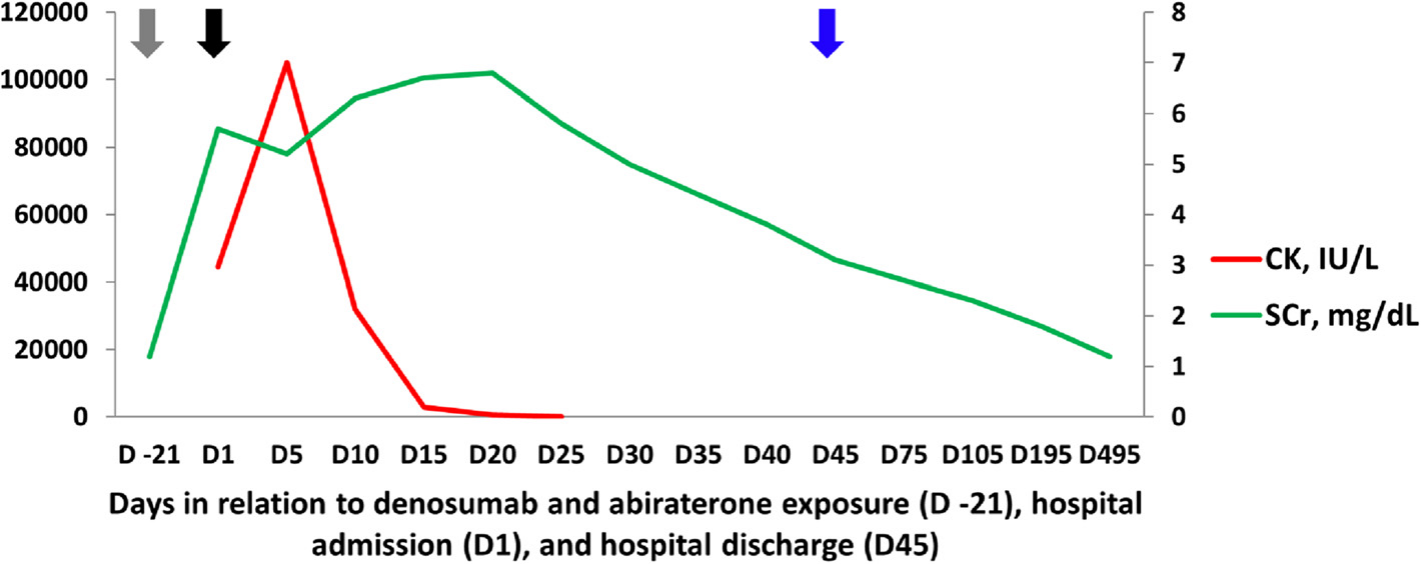
Time-related serum creatinine (SCr) and creatine kinase (CK) changes in relation to denosumab and abiraterone exposure (D −21), hospital admission (D1), and hospital discharge (D45). Gray arrow = denosumab 120 mg subcutaneous injection (single shot) and abiraterone started at 1 g orally per day. Black arrow = admission to the hospital, abiraterone stopped. Blue arrow = hospital discharge

**Fig. 2:**
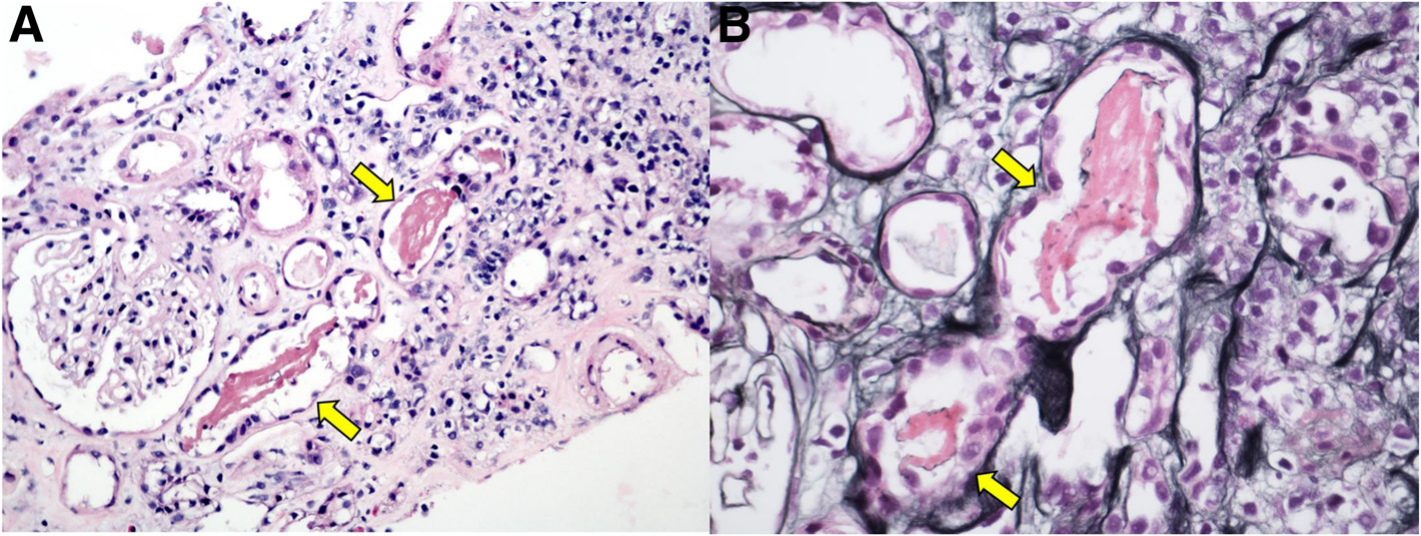
Kidney biopsy light microscopy. **a** Hematoxylin & eosin (X200) showing dark pink, filamentous tubular casts (yellow arrow); **b**) Jones’ silver stain (X400) showing dark pink, coarsely granular and filamentous tubular casts (yellow arrow)

**Fig. 3:**
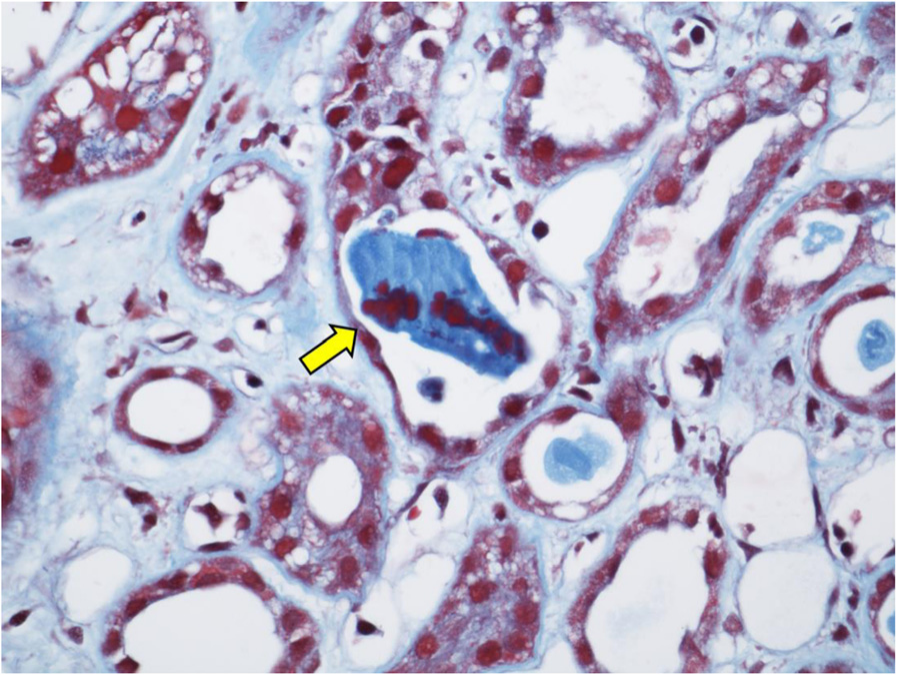
Kidney biopsy light microscopy showing tubular casts staining positively for myoglobin by immunohistochemistry (anti-myoglobin) (yellow arrow), (X400)

A 76-year-old Caucasian man with a history of type 2 diabetes, chronic kidney disease (CKD) stage 3A, essential hypertension, hypothyroidism, antiphospholipid antibody syndrome, prior cerebellar strokes, and prostate cancer (Gleason 10) with widespread metastasis to the bone presented with non-oliguric severe AKI 3 weeks after receiving simultaneous therapy with denosumab (120 mg subcutaneous injection once) and abiraterone (1 g per day orally). The patient had failed prior antineoplastic therapy with leuprolide acetate, bicalutamide, and nilutamide. On admission, his serum creatinine (SCr) was elevated at 5.7 mg/dL from a baseline of 1.2 mg/dL (Fig. 1). His active outpatient medications consisted of rosuvastatin (40 mg daily), benazepril, metoprolol tartrate, metformin, warfarin, low-dose prednisone (started concomitantly with abiraterone), and levothyroxine. The patient had been on statin therapy for more than 1 year and the dose had not been recently modified. The patient denied prior episodes of myopathies, rhabdomyolysis, or AKI. His physical exam was unremarkable. Further blood work showed hyperkalemia, mild metabolic acidosis, hypocalcemia, mild transaminemia (predominantly AST), and creatine kinase (CK) of 44,476 IU/L (Table 2). Urine studies revealed dipstick proteinuria (100 mg/dL), large dipstick blood, only a few normomorphic erythrocytes, and negative culture. All serologic work-up (ANA, PR3- and MPO-ANCA, anti-GBM antibodies, ENA panel, C3, C4, RF) and viral studies (HBV, HCV, CMV, EBV, Influenza A/B, Parainfluenza, Adenovirus, RSV) were negative or normal. Thyroid function tests were normal. Kidney sonogram and Doppler studies were negative for hydronephrosis and renal vein thrombosis, respectively. On admission, abiraterone therapy was discontinued and no further doses of denosumab were administered. Subsequently, the patient underwent kidney biopsy that was consistent with severe acute tubular injury with presence of myoglobin casts, confirming the diagnosis of rhabdomyolysis-induced AKI (Figs. 2 and 3). The patient responded well to intravenous isotonic fluids and discontinuation of denosumab, abiraterone, and rosuvastatin. CK levels normalized by day 25 of hospitalization and the SCr at the time of hospital discharge was 3.1 mg/dL (Fig. 1). After hospital discharge, the patient resumed all prior medications, including rosuvastatin, except for denosumab and abiraterone. His successive antineoplastic therapy consisted of enzalutamide. Kidney function returned to baseline 12 months after discharge and he had no recurrent episodes of rhabdomyolysis.

**Table 2:** Urinary and blood biochemical profiles at baseline, time of hospital admission, and at the time of peak creatine kinase (CK)

	Baseline (before admission)	At the time of admission	At the time of peak CK
*Blood studies*			
Sodium, mmol/L	135[135–145]	137	134 (L)
Potassium, mmol/L	4.6 [3.6–5.0]	6.2 (H)	3.7
Phosphorus, mg/dL	2.9 [2.4–4.5]	2.4	2.5
Calcium, mg/dL	10 [8.4–10.2]	8.6 (L)	5.7 (L)
Ionized calcium, mg/dL	-- [4.5–5.3]	--	3.4 (L)
Bicarbonate, mmol/L	29 [22–31]	21 (L)	13 (L)
Aspartate aminotransferase, U/L	29 [10–50]	354 (H)	1423 (H)
Alanine aminotransferase, U/L	19 [10–50]	125 (H)	330 (H)
Creatine kinase, IU/L	-- [39–308]	44,476 (H)	105,120 (H)
Lactate dehydrogenase, U/L	-- [135–225]	943 (H)	1135 (H)
Creatinine, mg/dL	1.2 [0.67–1.17]	5.7 (H)	5.2 (H)
eGFR, mL/min/1.73 m^2a^	59	10 (L)	11 (L)
*Urine studies*			
pH	-- [5.0–6.0]	6	6
Dipstick blood	-- [negative]	Large	Large
Dipstick protein, mg/dL	-- [negative]	100	100
Specific gravity	-- [1.008–1.030]	1.018	1.013
Red blood cells/HPF	-- [0–3/HPF]	15	35
White blood cells/HPF	-- [0–5/HPF]	<1	5
Squamous epithelial cells/HPF	-- [0–5/HPF]	10	<1
Leukocyte esterase	-- [negative]	Negative	Negative

[] = laboratory reference values; (H) = above laboratory reference value; (L) = below laboratory reference value;  --= not measured; ^a^eGFR = estimated glomerular filtration rate by MDRD study equation

## Conclusion

We present a case of a patient with biopsy-proven AKI caused by rhabdomyolysis 3 weeks after starting simultaneous treatment with denosumab and abiraterone. The patient was also taking rosuvastatin, a drug known to cause rhabdomyolysis [10]. However, his statin exposure was chronic and without reported recent dose adjustments. Most importantly, no other identifiable patient-specific triggers for statin-related rhabdomyolysis (e.g., decompensated hypothyroidism, liver disease, strenuous exercise, or recreational drug use) were identified upon interrogation and examination. In addition, his statin therapy was resumed after discharge with good tolerability thereafter. Nonetheless, it is not known if drug-drug interactions between the statin and denosumab or abiraterone could augment the risk for rhabdomyolysis in humans.

Denosumab is a human monoclonal antibody that neutralizes the receptor activator of nuclear factor kappa-B ligand (RANKL), inhibiting osteoclast-mediated bone resorption [11]. It is used to prevent bone metastasis and skeletal-related events in metastatic castration-resistant prostate cancer [12, 13]. Some of the common side effects of denosumab include back pain, cystitis, and upper respiratory tract infections. Serious adverse events such as osteonecrosis of the jaw have been reported in only 2 % of patients [14, 15]. Abiraterone is a CYP17A1 inhibitor that blocks the synthesis of androgens in prostate tumor cells, the testes, and adrenal glands. Its side effects are related to 17-alpha hydroxylase inhibition leading to a decrease in cortisol production and consequent increase in ACTH release, mineralocorticoid production, and ensuing hypokalemia, which itself is a risk factor for rhabdomyolysis [16, 17].

Prior to the development of abiraterone and its approval as an adrenal androgen synthesis inhibitor, ketoconazole was the only CYP17 inhibitor that was used to treat metastatic castration-resistant prostate cancer [18]. However, due to its actions on CYP3A and CYP24A1 and therefore the potential for drug-drug interactions, its use was limited to low-risk clinical scenarios [19]. A few cases of rhabdomyolysis with the concomitant use of ketoconazole and statin have been reported in the literature [20]. In this context, the risk of rhabdomyolysis is thought to be related to the inhibition of CYP3A by ketoconazole, precipitating a significant increase in the plasma concentration of statin. In contrast, abiraterone is known to act only on CYP17, with no apparent interference with statin metabolism. According to the product information, abiraterone metabolites inhibit the hepatic uptake transporter OATP1B1 *in vitro*, which may theoretically affect OATP1B1-mediated uptake of rosuvastatin, increase its levels, and therefore predispose to toxicity [21–23]. To our knowledge, there are no reported cases of rhabdomyolysis with either acute or chronic exposure to abiraterone, alone or in conjunction with rosuvastatin.

Over the past few years, genetic experiments have unveiled numerous functions of the RANKL/RANK system [11]. The best-known role of RANKL/RANK signaling is in bone. Genetic ablation of RANKL or RANK in mice results in a complete lack of osteoclasts, with consequent osteopetrosis and defective tooth eruption [11]. Indeed, inhibition of RANKL/RANK function in osteoclast formation, survival, and function underlies the therapeutic efficacy of denosumab in the treatment of osteoporosis [24]. RANKL also participates in the complex interplay of cancer cells with bone during bone metastasis, by promoting osteoclast formation and thus providing for establishment and growth of bone metastases [25]. These actions of RANKL underlie the efficacy of denosumab in ameliorating skeletal complications in metastatic cancer [12, 13]. However, animal studies have also revealed roles of this signaling pathway in extraskeletal tissues, such as in lymph node and immune cell development, and in mammary gland development and progestin-driven breast cancer [26]. Hypothalamic RANKL/RANK signaling also plays a key role in thermoregulation and fever [27]. Thus, roles for RANKL/RANK signaling have been found in bone, mammary tissue, the immune system, and CNS, suggesting pleiotropic effects on numerous cell and tissue types.

RANKL mRNA and protein are found in skeletal muscle [28]. However, its function in skeletal muscle is currently unknown, and awaits further studies with tissue-specific knockouts. Therefore, the possible effects of denosumab on skeletal muscle resulting in rhabdomyolysis remain speculative at this time. Possible mechanisms include a direct myopathic effect or alterations in calcium dynamics in the muscle cell, leading to an increase in intracellular calcium concentration and consequent rhabdomyolysis.

We report the first case of rhabdomyolysis leading to severe AKI in association with acute exposure to denosumab and abiraterone. From our case we are unable to determine whether rhabdomyolysis was caused by one of these two drugs, their mutual interaction, or their interaction with another commonly used drug, rosuvastatin. Nonetheless, this case exposes the need to carefully monitor patients exposed to denosumab and abiraterone to recognize potential severe adverse effects associated with these novel therapies. In addition, our report is hypothesis-generating to further investigate the effect of these drugs on muscle cells. In conclusion, we recommend monitoring for early signs of rhabdomyolysis in high-risk patients exposed to either denosumab or abiraterone, particularly if other identifiable risk factors are present (Table 1), such as in the case of our patient (e.g., advanced age, CKD, type 2 diabetes, hypothyroidism, and statin use).

## Consent

The patient was deceased at the time of preparation of this manuscript. Written informed consent was obtained from his next-of-kin for publication of this Case Report and any accompanying images.

## Abbreviations

ACTH: Adrenocorticotropic hormone
AKI: Acute kidney injury
ANA: Antinuclear antibody
AST: Aspartate aminotransferase
CK: Creatine kinase
CKD: Chronic kidney disease
CMV: Cytomegalovirus
CNS: Central nervous system
CYP: Cytochrome
EBV: Epstein-Barr virus
HBV: Hepatitis B virus
HCV: Hepatitis C virus
mRNA: Messenger RNA
RANKL: Receptor activator of nuclear factor kappa-B ligand
RSV: Respiratory syncytial virus
SCr: Serum creatinine
